# Exploring the role of retinal fluid as a biomarker for the management of diabetic macular oedema

**DOI:** 10.1038/s41433-023-02637-2

**Published:** 2023-07-21

**Authors:** Ramin Khoramnia, Quan Dong Nguyen, Peter J. Kertes, Laura Sararols Ramsay, Stela Vujosevic, Majid Anderesi, Franklin Igwe, Nicole Eter

**Affiliations:** 1 The David J. Apple International Laboratory for Ocular Pathology, Department of Ophthalmology, University of Heidelberg, Heidelberg, Germany; 2 Byers Eye Institute, Stanford University, Palo Alto, CA USA; 3 John and Liz Tory Eye Centre, Sunnybrook Health Sciences Centre, Toronto, ON Canada; 4 Department of Ophthalmology and Vision Sciences, University of Toronto, Toronto, ON Canada; 5 Hospital General de Catalunya, Barcelona, Spain; 6 Department of Biomedical, Surgical and Dental Sciences, University of Milan, Milan, Italy; 7 Eye Clinic, IRCCS MultiMedica, Milan, Italy; 8 Novartis Pharma AG, Basel, Switzerland; 9 Department of Ophthalmology, University of Münster Medical Center, Münster, Germany; 10 Present Address: OcuTerra Therapeutics, Basel, Switzerland

**Keywords:** Retinal diseases, Prognostic markers

## Abstract

Anti-VEGF therapies are associated with significant gains in visual acuity and fluid resolution in the treatment of diabetic macular oedema (DMO) and have become the standard of care. However, despite their efficacy, outcomes can be unpredictable, vary widely between individual eyes, and a large proportion of patients have persistent fluid following initial treatment, with a negative impact on visual outcomes. Anatomical parameters measured by optical coherence tomography (OCT), in addition to visual acuity, are key to monitoring treatment effectiveness and guiding retreatment decisions; however, existing guidelines on the management of DMO lack clear recommendations for interpretation of OCT parameters, or proposed thresholds of various markers to guide retreatment decisions. Although central subfield thickness (CSFT) has been widely used as a marker for retreatment decisions in clinical trials in DMO, and a reduction in CSFT has generally been shown to accompany improvements in best-corrected visual acuity with treatment, analyses of the relationship between these parameters show that the correlation is small to moderate. A more direct relationship can be seen between an increased magnitude of CSFT fluctuations over time and poorer visual acuity, suggesting that control of CSFT could be important in maximising visual outcomes. The relationship between visual outcomes and qualitatively assessed intraretinal fluid and subretinal fluid is also unclear, although quantitative assessments of fluid parameters suggest that untreated intraretinal fluid and subretinal fluid negatively impact visual outcomes. These findings highlight a need for clearer guidelines on the management of retinal fluid to improve visual outcomes for patients with DMO.

## Abstract (zh)

在糖尿病黄斑水肿(DMO)的治疗中, 抗VEGF治疗与视力的显著提高和视网膜积液的消除相关, 并已成为管理中的一个标准。然而, 抗VEGF治疗的有效性与视力预后未知, 个体之间差异很大, 大部分患者在初始治疗后有持续性积液, 对视力结局有负面影响。除了视力, 光学相干断层扫描 (OCT) 测量的解剖学参数是监测治疗效果和指导再治疗决策的关键, 然而, 现有的DMO治疗指南缺乏对OCT参数解释的明确建议, 也缺乏指导再治疗决策的各种标志物的建议性阈值。尽管黄斑中心凹下视网膜厚度(CSFT)已被广泛用作DMO临床试验中再治疗决策的标志, 并且 CSFT 的减少通常被视为伴随着治疗中最佳矫正视力的改善, 但是对这些参数之间关系的分析显示小到中等的相关性。随着时间推移, CSFT波动幅度的增加与视力下降之间存在更直接的关系, 这表明控制CSFT可以最大限度地改善视力。视力结局与定性评估的视网膜内液和视网膜下液之间的关系也不清楚, 尽管液体参数的定量评估表明未经治疗的视网膜内液和视网膜下液对视力结果有负面影响。这些发现强调了需要制定更明确的指导视网膜液管理指南, 以改善 DMO患者的视力结局。

## Introduction

The prevalence of diabetic macular oedema (DMO) was estimated to be 4% among people with diabetes in 2020, accounting for 18.83 million adults worldwide, with a projected increase to 28.61 million adults by 2045 [1]. DMO is a leading cause of visual impairment in patients with diabetes, particularly type 2 diabetes. Although DMO can occur at any age, a relatively high proportion of those affected come from a young, working-age population who are diagnosed on average at 50 years of age [2–5].

Patients with diabetes are at increased risk of several comorbidities, which can be complex to manage, and DMO is associated with a substantial treatment burden for patients, caregivers, and healthcare systems. The overall visit burden, time investment and complexity of therapy may limit capacity for anti-vascular endothelial growth factor (VEGF) treatment and reduce adherence [2, 6–9]. As a result, in real-world settings, the frequency of treatment received by patients can be lower than in clinical trials, which may lead to sub-optimal visual outcomes [10–13].

Another potential contributor to undertreatment in DMO is a lack of clear recommendations on retreatment in current treatment guidelines, particularly on the interpretation of retinal thickness or fluid on optical coherence tomography (OCT) images, resulting in variation in disease management between countries, regions, and private or public healthcare settings. This review aims to summarise existing knowledge on the role of retinal fluid in the pathophysiology of DMO and the short- and long-term impact on functional outcomes of unresolved fluid following treatment.

## Pathophysiology of DMO

**Fig. 1:**
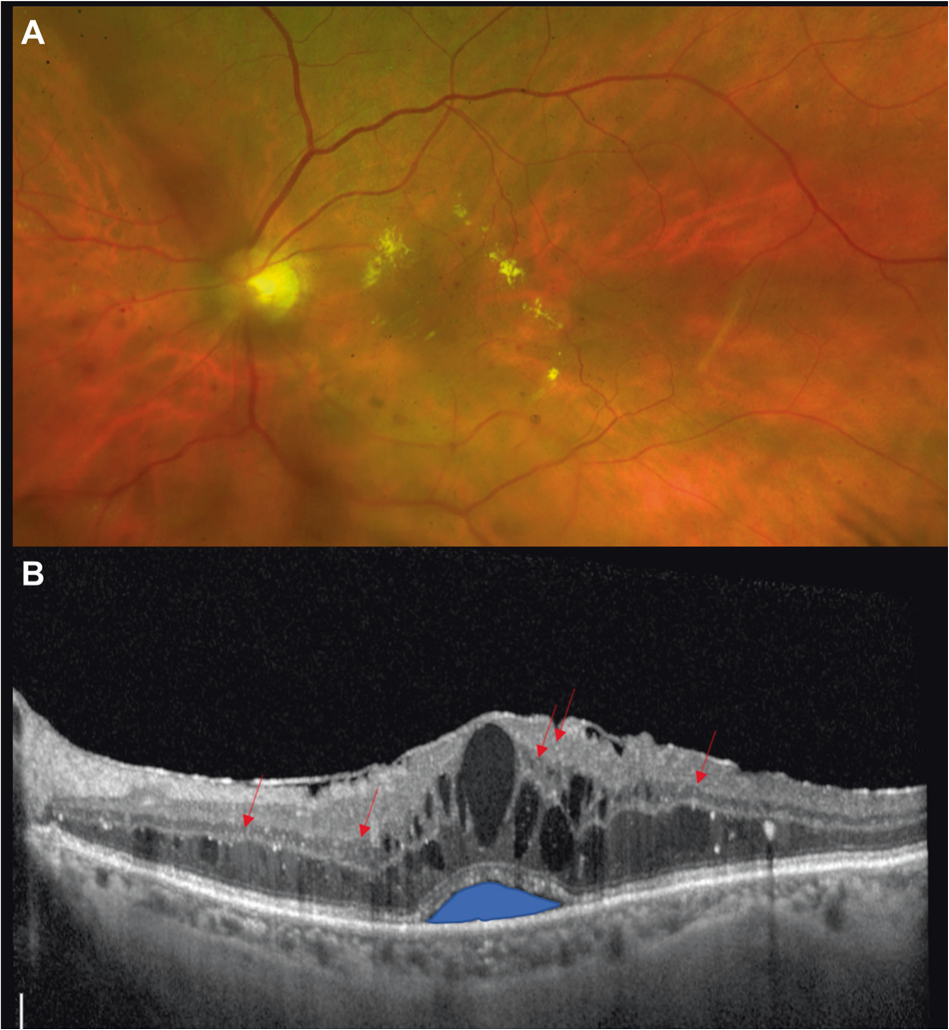
Pathophysiology of diabetic macular oedema. **A** Colour fundus photograph showing exudative diabetic macular oedema and moderate non-proliferative diabetic retinopathy. **B** Optical coherence tomography image showing diabetic macular oedema with sub-foveal neuroretina detachment (subretinal fluid), intraretinal cysts (intraretinal fluid) and hyperreflective spots showing activated microglial cells (indicated with arrows).

DMO can occur at any stage of diabetic retinopathy and is characterised by thickening of the macula leading to impairment of central visual function [14, 15]. Leakage of fluid and lipid-rich exudate into the retina, due to breakdown of the blood-retinal barrier, or focal leakage of microaneurysms distorts the retinal architecture, leading to thickening and reduced visual acuity if the centre of the macula is affected [14–17]. In addition to an increase in central subfield thickness (CSFT), morphologic hallmarks of DMO visualised by OCT are the accumulation of intraretinal fluid (IRF) and subretinal fluid (SRF), decreased reflectivity in the outer retinal layers, hyperreflective foci, and vitreomacular traction (Fig. 1) [14, 15, 18, 19].

Although both IRF and SRF are markers of DMO, IRF tends to be present in almost all patients at treatment initiation, while SRF is present in less than half [20–23]. SRF is generally considered to be associated with worse visual acuity than IRF at baseline and indicative of more severe disease, although it usually responds quickly to treatment [22–26]. Untreated fluid, irrespective of compartment, results in a gradual loss of vision over time and a delay in therapy initiation can lead to poorer treatment outcomes [27, 28].

## Current DMO management and impact of persistent oedema

Historically, the mainstay of DMO treatment was focal or grid laser photocoagulation; however, this has largely been supplanted with the availability of anti-VEGF therapies as a first-line treatment as, although laser can be effective in preventing DMO progression, the rate of vision improvements is low [29]. Certain indications remain for laser, however, including the vasogenic subform of DMO, eyes with DMO and central retinal thickness (CRT) less than 300 μm, or eyes with persisting vitreomacular adhesion. Subthreshold grid laser treatment can be useful in eyes with higher visual acuity affected by early diffuse DMO [30]. Anti-VEGF therapies are associated with significant gains in visual acuity and fluid resolution and have become the standard of care for DMO treatment [31–33]. Intravitreal corticosteroids are another, less common treatment option for patients who are non- or incompletely responsive to anti-VEGFs [34–36].

**Fig. 2:**
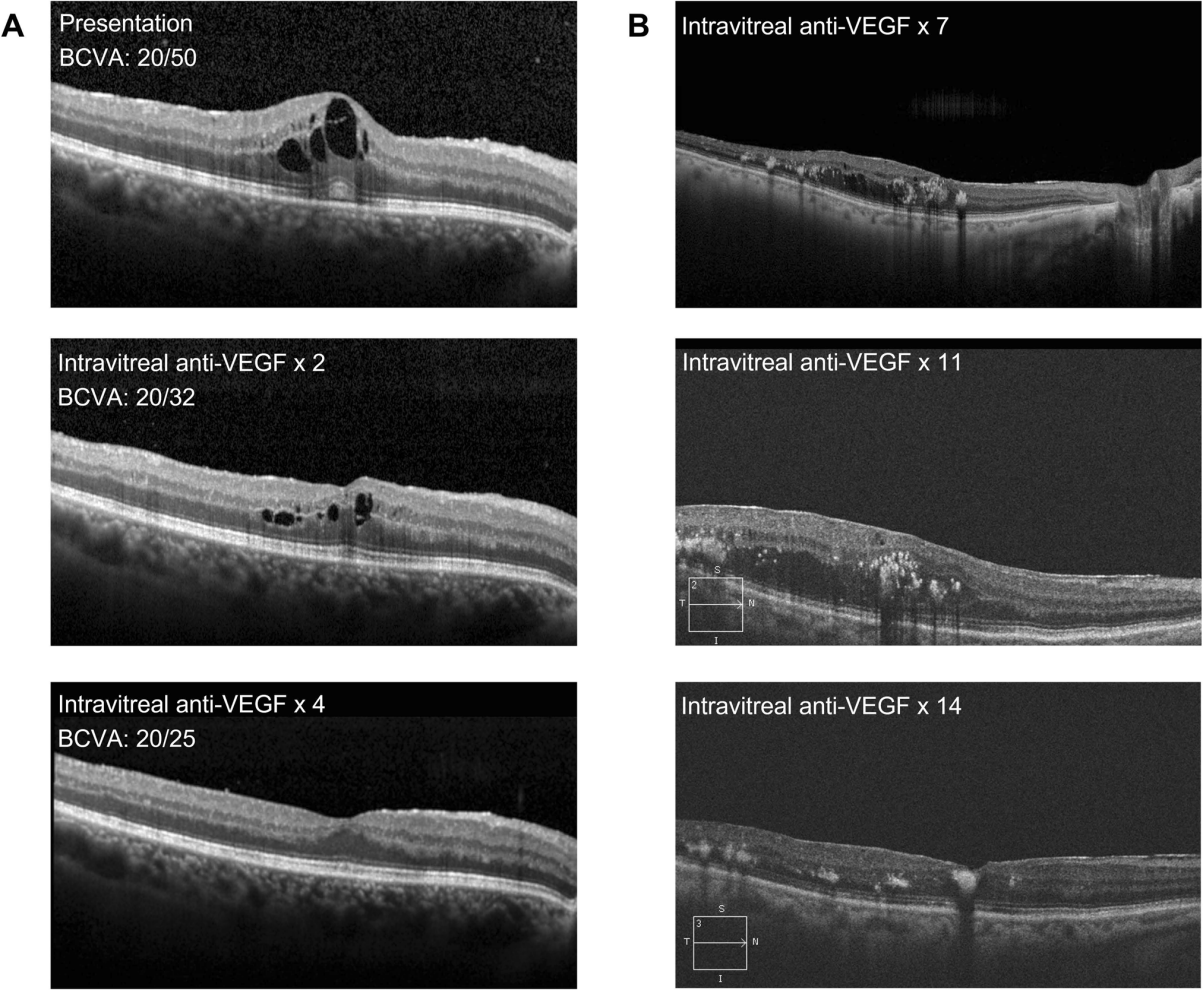
Optical coherence tomography images showing response to anti-vascular endothelial growth factor treatment in patients with diabetic macular oedema. **A** An example of ‘good’ response with resolution of retinal fluid after 4 monthly anti-vascular endothelial growth factor injections. **B** Delayed response to monthly anti-vascular endothelial growth factor treatment.

Despite the efficacy of available treatments for DMO, outcomes can be unpredictable and vary widely between individual eyes. In addition to visual acuity, anatomical parameters measured on OCT are key to monitoring treatment effectiveness and guiding treatment decisions; current guidelines on DMO management recommend that retreatment decisions should be based on a combination of visual acuity and OCT findings [30, 37, 38]. However, the association between anatomic parameters and visual outcomes is not fully defined and there is a lack of clear guidance on the interpretation of OCT parameters in relation to treatment decisions or on a threshold for OCT markers to denote treatment response. In a clinical setting, retreatment decisions are often qualitative rather than quantitative and consider factors such as the pattern of DMO, involvement of the centre of the fovea, integrity of the inner and outer retinal layers, presence and quantity of hyperreflective foci, and extension/reflectivity of retinal cysts. Other considerations are visual acuity of the contralateral eye, the general systemic condition of the patient, and the ability of the patient to attend frequent appointments. Examples of response to anti-VEGF treatment are shown in Fig. 2.

Although CSFT has been widely used as a marker for retreatment decisions in clinical trials in DMO, often in combination with visual acuity criteria, there is a large degree of variation in re-treatment thresholds applied. In trials of adaptive treatment regimens, such as pro re nata or treat-and-extend, retinal thickness thresholds for retreatment ranged from 225 to 325 µm, with or without additional criteria such as a change of >10% from the previous visit, the absence of stable measurements over consecutive visits or associated SRF and/or IRF [28, 39–44].

A lack of response to treatment is associated with poor visual outcomes for patients with DMO. In a post hoc analysis of Protocol I, eyes receiving ranibizumab plus prompt or deferred laser with higher average levels of oedema (calculated as excess CRT [≥250 µm] averaged over 52 weeks) gained 9.3 fewer Early Treatment Diabetic Retinopathy Study (ETDRS) letters than those with lower levels at the end of Year 3. Patients with the longest duration of persistent oedema (calculated as the number of study visits with CRT ≥ 250 µm during the first 52 weeks of treatment) gained 4.4 fewer letters than those with the least persistent oedema [45]. The negative correlation between duration and extent of oedema and visual outcomes was suggested to be due to photoreceptor degeneration [45, 46].

Rates of persistent DMO in clinical studies (based on CSFT ≥ 250 µm) can be in the range of 20–60% after 2 years of treatment [47–49]. A secondary analysis of Protocol T showed rates of persistent DMO at Week 24 (defined as CSFT ≥ 250 µm at each completed study visit through Week 24) of 31.6% for aflibercept, 41.5% for ranibizumab and 65.6% for bevacizumab. Among these patients, rates of chronic persistent DMO (defined as failure to achieve CSFT < 250 µm and a reduction in CSFT of at least 10% relative to the Week 24 visit on at least two consecutive visits) at 2 years were 44.2% with aflibercept, 54.5% with ranibizumab and 68.2% with bevacizumab [50].

In clinical practice, where anti-VEGF administration tends to be less frequent than in clinical trials, response to treatment can be even lower. A retrospective chart review of patients receiving anti-VEGF therapy at 10 sites in the US assessed the proportion of patients with best-corrected visual acuity (BCVA) of 20/40 or better combined with CRT ≤ 250 µm on time domain (TD)-OCT or ≤300 µm on spectral domain (SD)-OCT over 12 anti-VEGF injections. For injections 1–9, BCVA of 20/40 or better was achieved by 52–62% of patients and the defined CRT threshold was achieved by 26–34% of patients. The proportion of patients achieving both BCVA and CRT endpoints ranged from only ~20–40% over all 12 injections [51].

A post hoc analysis of eyes with persistent DMO from Protocol I (approximately 40% of patients in that study) showed that eyes receiving ranibizumab with prompt or deferred laser with chronic persistent DMO (failure to achieve CSFT <250 μm and a ≥10% reduction from the 24-week visit on ≥2 consecutive study visits) at 3 years had worse visual acuity than those without, highlighting the importance of optimising anatomical outcomes for these patients [47].

Factors associated with an increased likelihood of persistent DMO, including high baseline CSFT and limited early visual and morphologic responses, have been shown to be predictive of long-term outcomes; therefore, the identification of a simple biomarker of treatment response would further support the management of patients with DMO [52–55].

## Relationship between OCT markers of retinal fluid and visual outcomes

Generally, in clinical studies in DMO, including treatment with anti-VEGF, laser, or corticosteroids, BCVA improvement in response to treatment is accompanied by a reduction in retinal thickness [32, 39, 49, 56–58]. However, the nature of the relationship between visual outcomes and retinal thickness and whether a direct association exists is unclear. CSFT is the most used OCT biomarker in DMO management based on the association between central involvement of DMO and visual acuity, and greater reproducibility compared with other measures of retinal thickness such as foveal centre point thickness [18, 59].

Post hoc analyses of multiple clinical trials (Protocol T, TREX-DME, and DAVE trials using anti-VEGF therapies; TYBEE and HULK trials of corticosteroids; and the Protocol I trial of focal/grid laser) showed a correlation between CSFT and BCVA at baseline and following treatment, or in change in CSFT and BCVA over time. Furthermore, the correlation increased in groups with higher CSFT when stratified by baseline levels. However, the correlations were small to moderate at best, with changes in CSFT accounting for a small proportion of the total changes in visual acuity, leading the authors to conclude that the findings did not support CSFT as a surrogate for BCVA [60–63]. A further post hoc analysis of patients receiving ranibizumab and/or laser photocoagulation in the RESTORE/RESTORE-extension studies showed a low correlation between CSFT and BCVA at baseline, which was lost over time [64]. Low to moderate correlations were also seen in a number of smaller retrospective cohort analyses or consecutive case series, although further small studies showed a lack of significant correlation [23, 65–71].

While a strong correlation may not exist between CSFT and BCVA at discrete timepoints or based on a specific difference between two timepoints, a more recent post hoc analysis of data from the Protocol T and Protocol V studies and a retrospective cohort study at the Cleveland Clinic (Cleveland, OH, USA) have shown a significant correlation between increased fluctuations in CSFT over the course of anti-VEGF treatment and worse visual outcomes [72, 73]. Based on data from the Protocol T and V trials (in eyes receiving anti-VEGF therapy or focal/grid laser), there was a difference of 1.61 and 3.04 ETDRS letters, respectively, between patients in quartiles with the highest and lowest CSFT fluctuations (measured as the standard deviation [SD] of a patient’s CSFT over treatment) after 12 months [72]. In the Cleveland Clinic study of eyes receiving anti-VEGF therapy, with the same measure of fluctuation, there was a mean difference of 6.87 ETDRS letters over 12 months per 100 µm CSFT SD and the difference between the quartiles with the highest and lowest fluctuations was 9.7 ETDRS letters after 12 months [73]. These findings suggest that CSFT fluctuations over time may be prognostic of visual outcomes in patients with DMO treated with anti-VEGFs.

CSFT is reflective of a number of parameters and pathophysiological processes, which is assumed to be based to a large degree on the contribution of retinal fluid. However, a study using a deep-learning approach to assess the correlation between CSFT and IRF or SRF volume showed that CSFT is only partly driven by IRF, and not SRF, volume at baseline and during anti-VEGF treatment and is therefore not a direct measure of exudative activity. Using SD-OCT images from 656 patients from Protocol T, a moderate correlation was seen between CSFT at baseline and IRF alone (0.688) or IRF and SRF combined (0.753), whereas the correlation between CSFT and SRF was low (0.408). Under anti-VEGF therapy, the correlation between CSFT and IRF alone (0.797) and IRF and SRF combined (0.805) increased to high, whereas the correlation between CSFT and SRF alone decreased (0.082) [24]. This and other recent studies using artificial intelligence approaches suggest that retinal fluid volume may be a more reliable biomarker for the monitoring of DMO than CSFT [22, 24, 26].

In terms of the impact of individual fluid types on visual outcomes, a post hoc analysis of the RESTORE study showed that patients treated with ranibizumab, laser photocoagulation, or both, with baseline intraretinal cystoid fluid (IRC) height ≤380 µm had better BCVA than those with IRC > 380 µm at both baseline and Month 12; IRC height at baseline was also a better predictor of outcomes than CSFT. However, in patients followed up through the RESTORE-extension study, there was no significant difference between IRC groups at Month 36 [64]. A moderate negative correlation between IRC height and BCVA was also seen in a retrospective case series of 66 patients that had not received treatment with anti-VEGFs in the prior 3 months or steroids in the prior 6 months [69]. Conversely, a retrospective cohort study of 119 patients receiving ranibizumab showed no significant correlation between IRC and BCVA [68], while a study of 159 patients receiving bevacizumab showed that baseline ‘severe IRF’ (≥50% of the linear scan of the horizontal raster scan of the fovea) was significantly more likely in eyes gaining 3 or more lines of BCVA compared with eyes that lost 3 or more lines. Similar findings were also seen in patients with more moderate vision changes (gain or loss of ≥1 line of vision). The authors suggested that eyes with less IRF have more baseline macular ischaemia and thus less room for improvement, or that greater IRF at baseline may simply allow for a more significant reduction with anti-VEGF therapy, resulting in improved BCVA [23].

In a sub-analysis of the RISE and RIDE trials, SRF at baseline was predictive of better visual outcomes following treatment with ranibizumab [25]. A retrospective cohort study of eyes treated with an intravitreal dexamethasone implant also showed that SRF at baseline was predictive of better visual outcomes following treatment with dexamethasone implants, with treatment-naïve eyes showing a better response than refractory eyes [71]. The VIVID-DME and VISTA-DME studies, however, showed similar BCVA gains at Weeks 52 and 100 for patients treated with aflibercept irrespective of baseline SRF but BCVA loss of approximately 2 letters for those with baseline SRF treated with laser compared with a gain of greater than 2 letters for those without baseline SRF [21]. A post hoc analysis of the RESTORE/RESTORE-extension studies also showed worse BCVA outcomes in patients with baseline SRF at 12 months following laser treatment, while patients treated with ranibizumab with baseline SRF had better BCVA outcomes than those without and patients receiving combination treatment of ranibizumab plus laser had similar outcomes regardless of SRF presence or absence [64].

Many analyses on the association between fluid and visual outcomes rely on qualitative rather than quantitative assessment of fluid parameters, which may not provide a complete reflection of this relationship e.g. measures of IRC height may only take the highest cyst into account and only a moderate correlation exists between SRF fluid volume and fluid height at baseline and during treatment [24]. A volumetric analysis of SD-OCT images from eyes receiving anti-VEGF treatment in the Protocol T trial using a deep-learning algorithm showed significantly higher IRF and SRF in eyes with worse BCVA at baseline, and for IRF after a year of treatment. SRF had a stronger association with BCVA than IRF, with every 10 nL reduction in fluid in the central fovea translating to an improvement in ETDRS letter score of 0.34 and 0.15, respectively, during Year 1. Although the presence of SRF was associated with worse BCVA and higher IRF volume at baseline, and with greater improvements in BCVA at each assessment through 12 months of treatment, there was no difference in BCVA or IRF between eyes with or without SRF after 12 months [22]. A retrospective cohort study using a similar approach to quantify IRF, SRF, and total retinal fluid showed that presence of IRF or SRF after 12 months of anti-VEGF treatment was associated with significantly lower BCVA. In a comparison of fluid volume quartiles (quartile 1 having the lowest volume), IRF alone was associated with a significant difference in BCVA for the second, third, and fourth fluid quartiles of −2.23, −4.41, and −8.63 letters, respectively, at 1 year; SRF was associated with a significant difference in the fourth quartile only (of −5.38 letters); a combination of the two was associated with significant differences in the third and fourth quartile, of −4.79 and −8.85 letters, respectively [26].

In addition to CSFT and retinal fluid, ellipsoid zone integrity and, in particular, the relative ellipsoid zone reflectivity ratio has been identified as a potential biomarker for therapy surveillance and prediction of visual acuity outcomes. A longitudinal study showed a correlation between elipsoid zone integrity and visual acuity from baseline to Year 5, demonstrating the relationship beyond 1 year of therapy [74]. Another study assessed semi-automated quantification of retinal and choroidal biomarkers on OCT in patients with diabetic retinopathy complicated by macular oedema. All three OCT biomarkers evaluated—number of hyperreflective foci, ellipsoid zone reflectivity ratio, and choroidal vascularity index—have been suggested to correlate with visual acuity change or treatment outcomes. The study demonstrated excellent reproducibility of these biomarkers on SD-OCT with and without enhanced depth imaging mode, regardless of the presence of macular oedema [75]. A further retrospective review of visual outcomes in DMO patients receiving anti-VEGF therapy showed the extent of ellipsoid zone and external limiting membrane disruption at 12 months is negatively correlated with the area and number of intraretinal cysts at baseline [76].

## Conclusions

In current practice, anti-VEGFs are the first-line treatment option for DMO patients. However, there is a large proportion of patients with persistent fluid in the real world despite initial anti-VEGF treatment. This persistent oedema is associated with negative visual outcomes, highlighting an unmet need for a significant cohort of patients and a gap in existing treatment guidelines in terms of clear recommendations relating to retinal fluid in disease management. CSFT has been widely adopted as a marker of treatment response, although various analyses suggest the association between CSFT and visual outcomes is moderate at best. While CSFT itself may not be strongly associated with visual outcomes, CSFT fluctuations seem to be a good correlate, suggesting that control of CSFT is important in maximising visual outcomes and CSFT fluctuations may be considered by clinicians when making retreatment decisions. Additionally, fluid parameters play a role in assessing the effectiveness of treatment, retreatment decisions, and therefore ability to extend treatment intervals. Studies using quantitative assessments of fluid parameters suggest that untreated IRF and SRF are associated with a negative impact on visual outcomes, which may correlate with fluid volume in the case of IRCs. A more stringent approach to the treatment of retinal fluid and clearer recommendations on the integration of fluid parameters into retreatment decisions may improve visual outcomes for patients with DMO.

## Methodology

**Search terms:**

‘Diabetic macular o/edema’ OR ‘clinically significant macular o/edema’

AND

‘treatment guideline / recommendation’; ‘retinal fluid’; ‘intraretinal fluid’; ‘intraretinal cysts’; ‘intraretinal cystoid o/edema’; ‘cystoid o/edema’; ‘cystoid macular o/edema’; ‘subretinal fluid’; ‘subretinal pigment epithelial fluid’; ‘serous retinal detachment’; ‘central retinal thickness’; ‘central subfield thickness’; ‘fluid management’; ‘optical coherence tomography’; ‘spectral domain-optical coherence tomography’; ‘optical coherence tomography-angiography’.

**Search criteria:** English language

**Databases searched:** Embase and Medline

## References

[1] Teo ZL, Tham YC, Yu M, Chee L, Rim TH, Cheung N (2021). Global prevalence of diabetic retinopathy and projection of burden through 2045: Systematic review and meta-analysis. Ophthalmology.

[2] Spooner KL, Guinan G, Koller S, Hong T, Chang AA (2019). Burden Of treatment among patients undergoing intravitreal injections for diabetic macular oedema in Australia. Diabetes Metab Syndr Obes.

[3] Petrella RJ, Blouin J, Davies B, Barbeau M (2012). Prevalence, demographics, and treatment characteristics of visual impairment due to diabetic macular edema in a representative Canadian cohort. J Ophthalmol.

[4] Liew G, Michaelides M, Bunce C (2014). A comparison of the causes of blindness certifications in England and Wales in working age adults (16–64 years), 1999–2000 with 2009–2010. BMJ Open.

[5] Lee R, Wong TY, Sabanayagam C (2015). Epidemiology of diabetic retinopathy, diabetic macular edema and related vision loss. Eye Vis.

[6] Lu AJ, Chen AJ, Hwang V, Law PY, Stewart JM, Chao DL (2019). Analysis of patient-reported barriers to diabetic retinopathy follow-up. Ophthalmic Surg Lasers Imaging Retina.

[7] Rose MA, Vukicevic M, Koklanis K, Rees G, Sandhu S, Itsiopoulos C (2019). Experiences and perceptions of patients undergoing treatment and quality of life impact of diabetic macular edema: a systematic review. Psychol Health Med.

[8] Wallick CJ, Hansen RN, Campbell J, Kiss S, Kowalski JW, Sullivan SD (2015). Comorbidity and health care resource use among commercially insured non-elderly patients with diabetic macular edema. Ophthalmic Surg Lasers Imaging Retina.

[9] Weiss M, Sim DA, Herold T, Schumann RG, Liegl R, Kern C (2018). Compliance and adherence of patients with diabetic macular edema to intravitreal anti-vascular endothelial growth factor therapy in daily practice. Retina..

[10] Kiss S, Liu Y, Brown J, Holekamp NM, Almony A, Campbell J (2014). Clinical utilization of anti-vascular endothelial growth-factor agents and patient monitoring in retinal vein occlusion and diabetic macular edema. Clin Ophthalmol.

[11] Massin P, Creuzot-Garcher C, Kodjikian L, Girmens JF, Delcourt C, Fajnkuchen F (2019). Real-world outcomes with ranibizumab 0.5 mg in patients with visual impairment due to diabetic macular edema: 12-Month Results from the 36-Month BOREAL-DME Study. Ophthalmic Res.

[12] Stefanickova J, Cunha-Vaz J, Ulbig M, Pearce I, Fernandez-Vega Sanz A, Theodossiadis P (2018). A noninterventional study to monitor patients with diabetic macular oedema starting treatment with ranibizumab (POLARIS). Acta Ophthalmol.

[13] Ziemssen F, Wachtlin J, Kuehlewein L, Gamulescu MA, Bertelmann T, Feucht N (2018). Intravitreal ranibizumab therapy for diabetic macular edema in routine practice: Two-year real-life data from a non-interventional, multicenter study in Germany. Diabetes Ther.

[14] Das A (2016). Diabetic Retinopathy: Battling the global epidemic. Investig Ophthalmol Vis Sci.

[15] Waheed NK. Diabetic macular edema. In: Goldman DR, Waheed NK, Duker JS, editors. Atlas of retinal OCT: optical coherence tomography. The Netherlands: Elsevier Inc.; 2018.

[16] Boyer DS, Hopkins JJ, Sorof J, Ehrlich JS (2013). Anti-vascular endothelial growth factor therapy for diabetic macular edema. Ther Adv Endocrinol Metab.

[17] Stewart MW (2012). Anti-vascular endothelial growth factor drug treatment of diabetic macular edema: the evolution continues. Curr Diabetes Rev.

[18] Buabbud JC, Al-latayfeh MM, Sun JK (2010). Optical coherence tomography imaging for diabetic retinopathy and macular edema. Curr Diab Rep.

[19] Vujosevic S, Torresin T, Berton M, Bini S, Convento E, Midena E (2017). Diabetic macular edema with and without subfoveal neuroretinal detachment: Two different morphologic and functional entities. Am J Ophthalmol.

[20] Korobelnik JF, Daien V, Faure C, Tadayoni R, Giocanti-Auregan A, Dot C (2020). Real-world outcomes following 12 months of intravitreal aflibercept monotherapy in patients with diabetic macular edema in France: results from the APOLLON study. Graefes Arch Clin Exp Ophthalmol.

[21] Korobelnik JF, Lu C, Katz TA, Dhoot DS, Loewenstein A, Arnold J (2019). Effect of baseline subretinal fluid on treatment outcomes in VIVID-DME and VISTA-DME studies. Ophthalmol Retin.

[22] Roberts PK, Vogl WD, Gerendas BS, Glassman AR, Bogunovic H, Jampol LM (2020). Quantification of fluid resolution and visual acuity gain in patients with diabetic macular edema using deep learning: a post hoc analysis of a randomized clinical trial. JAMA Ophthalmol.

[23] Itoh Y, Petkovsek D, Kaiser PK, Singh RP, Ehlers JP (2016). Optical coherence tomography features in diabetic macular edema and the impact on anti-VEGF response. Ophthalmic Surg Lasers Imaging Retina.

[24] Pawloff M, Bogunovic H, Gruber A, Michl M, Riedl S, Schmidt-Erfurth U (2022). A systematic correlation of central subfield thickness (CSFT) with retinal fluid volumes quantified by deep learning in the major exudative macular diseases. Retina..

[25] Sophie R, Lu N, Campochiaro PA (2015). Predictors of functional and anatomic outcomes in patients with diabetic macular edema treated with ranibizumab. Ophthalmology..

[26] Kalur A, Iyer AI, Muste JC, Talcott KE, Singh RP (2022). Impact of retinal fluid in patients with diabetic macular edema treated with anti-VEGF in routine clinical practice.. Can J Ophthalmol..

[27] Brown DM, Nguyen QD, Marcus DM, Boyer DS, Patel S, Feiner L (2013). Long-term outcomes of ranibizumab therapy for diabetic macular edema: the 36-month results from two phase III trials: RISE and RIDE. Ophthalmology..

[28] Massin P, Bandello F, Garweg JG, Hansen L, Harding SP, Larsen M (2010). Safety and efficacy of ranibizumab in diabetic macular edema (RESOLVE Study): a 12-month, randomized, controlled, double-masked, multicenter phase II study. Diabetes Care.

[29] Early Treatment Diabetic Retinopathy Study Research Group. (1985). Photocoagulation for diabetic macular edema: early treatment diabetic retinopathy study report number 1. Arch Ophthalmol.

[30] Schmidt-Erfurth U, Garcia-Arumi J, Bandello F, Berg K, Chakravarthy U, Gerendas BS (2017). Guidelines for the management of diabetic macular edema by the European Society of Retina Specialists (EURETINA). Ophthalmologica..

[31] Elman MJ, Aiello LP, Beck RW, Bressler NM, Bressler SB, Diabetic Retinopathy Clinical Research Network (2012). Intravitreal ranibizumab for diabetic macular edema with prompt versus deferred laser treatment: three-year randomized trial results. Ophthalmology.

[32] Korobelnik JF, Do DV, Schmidt-Erfurth U, Boyer DS, Holz FG, Heier JS (2014). Intravitreal aflibercept for diabetic macular edema. Ophthalmology.

[33] Rajendram R, Fraser-Bell S, Kaines A, Michaelides M, Hamilton RD, Esposti SD (2012). A 2-year prospective randomized controlled trial of intravitreal bevacizumab or laser therapy (BOLT) in the management of diabetic macular edema: 24-month data: report 3. Arch Ophthalmol.

[34] Boyer DS, Yoon YH, Belfort R, Bandello F, Maturi RK, Augustin AJ (2014). Three-year, randomized, sham-controlled trial of dexamethasone intravitreal implant in patients with diabetic macular edema. Ophthalmology.

[35] Augustin AJ, Bopp S, Fechner M, Holz F, Sandner D, Winkgen AM (2020). Three-year results from the Retro-IDEAL study: Real-world data from diabetic macular edema (DME) patients treated with ILUVIEN((R)) (0.19 mg fluocinolone acetonide implant). Eur J Ophthalmol.

[36] Augustin AJ, Bopp S, Fechner M, Holz FG, Sandner D, Winkgen AM (2022). The impact of vitrectomy on outcomes achieved with 0.19 mg fluocinolone acetonide implant in patients with diabetic macular edema. Eur J Ophthalmol.

[37] Flaxel CJ, Adelman RA, Bailey ST, Fawzi A, Lim JI, Vemulakonda GA (2020). Diabetic retinopathy preferred practice pattern®. Ophthalmology..

[38] Wong TY, Sun J, Kawasaki R, Ruamviboonsuk P, Gupta N, Lansingh VC (2018). Guidelines on Diabetic Eye Care: The International Council of Ophthalmology recommendations for screening, follow-up, referral, and treatment based on resource settings. Ophthalmology..

[39] Wells JA, Glassman AR, Ayala AR, Jampol LM, Aiello LP, Diabetic Retinopathy Clinical Research Network (2015). Aflibercept, bevacizumab, or ranibizumab for diabetic macular edema. N Engl J Med.

[40] Ehlers JP, Wang K, Singh RP, Babiuch AS, Schachat AP, Yuan A (2018). A prospective randomized comparative dosing trial of ranibizumab in bevacizumab-resistant diabetic macular edema: The REACT study. Ophthalmol Retina.

[41] Michaelides M, Kaines A, Hamilton RD, Fraser-Bell S, Rajendram R, Quhill F (2010). A prospective randomized trial of intravitreal bevacizumab or laser therapy in the management of diabetic macular edema (BOLT study) 12-month data: report 2. Ophthalmology..

[42] Nguyen QD, Shah SM, Khwaja AA, Channa R, Hatef E, Do DV (2010). Two-year outcomes of the ranibizumab for edema of the mAcula in diabetes (READ-2) study. Ophthalmology..

[43] Payne JF, Wykoff CC, Clark WL, Bruce BB, Boyer DS, Brown DM (2017). Randomized trial of treat and extend ranibizumab with and without navigated laser for diabetic macular edema: TREX-DME 1 year outcomes. Ophthalmology..

[44] Pearce I, Banerjee S, Burton BJ, Chakravarthy U, Downey L, Gale RP (2015). Ranibizumab 0.5 mg for diabetic macular edema with bimonthly monitoring after a phase of initial treatment: 18-month, multicenter, phase IIIB RELIGHT study. Ophthalmology..

[45] Sadda SR, Campbell J, Dugel PU, Holekamp NM, Kiss S, Loewenstein A (2020). Relationship between duration and extent of oedema and visual acuity outcome with ranibizumab in diabetic macular oedema: a post hoc analysis of Protocol I data. Eye.

[46] Murakami T, Nishijima K, Akagi T, Uji A, Horii T, Ueda-Arakawa N (2012). Optical coherence tomographic reflectivity of photoreceptors beneath cystoid spaces in diabetic macular edema. Investig Ophthalmol Vis Sci.

[47] Bressler SB, Ayala AR, Bressler NM, Melia M, Qin H, Ferris FL (2016). Persistent macular thickening after ranibizumab treatment for diabetic macular edema with vision impairment. JAMA Ophthalmol.

[48] Elman MJ, Bressler NM, Qin H, Beck RW, Ferris FL, Friedman SM (2011). Expanded 2-year follow-up of ranibizumab plus prompt or deferred laser or triamcinolone plus prompt laser for diabetic macular edema. Ophthalmology.

[49] Nguyen QD, Brown DM, Marcus DM, Boyer DS, Patel S, Feiner L (2012). Ranibizumab for diabetic macular edema: results from 2 phase III randomized trials: RISE and RIDE. Ophthalmology.

[50] Bressler NM, Beaulieu WT, Glassman AR, Blinder KJ, Bressler SB, Jampol LM (2018). Persistent macular thickening following intravitreous aflibercept, bevacizumab, or ranibizumab for central-involved diabetic macular edema with vision impairment: A secondary analysis of a randomized clinical trial. JAMA Ophthalmol.

[51] Blinder KJ, Dugel PU, Chen S, Jumper JM, Walt JG, Hollander DA (2017). Anti-VEGF treatment of diabetic macular edema in clinical practice: effectiveness and patterns of use (ECHO Study Report 1). Clin Ophthalmol.

[52] Dugel PU, Campbell JH, Kiss S, Loewenstein A, Shih V, Xu X (2019). Association between early anatomic response to anti-vascular endothelial growth factor therapy and long-term outcome in diabetic macular edema: An independent analysis of Protocol I study data. Retina..

[53] Gonzalez VH, Campbell J, Holekamp NM, Kiss S, Loewenstein A, Augustin AJ (2016). Early and long-term responses to anti-vascular endothelial growth factor therapy in diabetic macular edema: Analysis of Protocol I data. Am J Ophthalmol.

[54] Halim MS, Afridi R, Hasanreisoglu M, Hassan M, Ibrahim-Ahmed M, Do DV (2021). Differences in the characteristics of subjects achieving complete, partial, or no resolution of macular edema in the READ-3 study. Graefes Arch Clin Exp Ophthalmol.

[55] Sivaprasad S, Crosby-Nwaobi R, Heng LZ, Peto T, Michaelides M, Hykin P (2013). Injection frequency and response to bevacizumab monotherapy for diabetic macular oedema (BOLT Report 5). Br J Ophthalmol.

[56] Brown DM, Emanuelli A, Bandello F, Barranco JJE, Figueira J, Souied E (2022). KESTREL and KITE: 52-week results from two phase III pivotal trials of brolucizumab for diabetic macular edema. Am J Ophthalmol.

[57] Elman MJ, Aiello LP, Beck RW, Bressler NM, Bressler SB, Diabetic Retinopathy Clinical Research Network (2010). Randomized trial evaluating ranibizumab plus prompt or deferred laser or triamcinolone plus prompt laser for diabetic macular edema. Ophthalmology.

[58] Mitchell P, Bandello F, Schmidt-Erfurth U, Lang GE, Massin P, Schlingemann RO (2011). The RESTORE study: ranibizumab monotherapy or combined with laser versus laser monotherapy for diabetic macular edema. Ophthalmology..

[59] Davis MD, Bressler SB, Aiello LP, Bressler NM, Browning DJ, Flaxel CJ (2008). Comparison of time-domain OCT and fundus photographic assessments of retinal thickening in eyes with diabetic macular edema. Investig Ophthalmol Vis Sci.

[60] Bressler NM, Odia I, Maguire M, Glassman AR, Jampol LM, MacCumber MW (2019). Association between change in visual acuity and change in central subfield thickness during treatment of diabetic macular edema in participants randomized to aflibercept, bevacizumab, or ranibizumab: a post hoc analysis of the Protocol T randomized clinical trial. JAMA Ophthalmol.

[61] Ciulla TA, Kapik B, Grewal DS, Ip MS (2021). Visual acuity in retinal vein occlusion, diabetic, and uveitic macular edema: Central subfield thickness and ellipsoid zone analysis. Ophthalmol Retina.

[62] Browning DJ, Glassman AR, Aiello LP, Beck RW, Brown DM, Diabetic Retinopathy Clinical Research Network (2007). Relationship between optical coherence tomography-measured central retinal thickness and visual acuity in diabetic macular edema. Ophthalmology.

[63] Ou WC, Brown DM, Payne JF, Wykoff CC (2017). Relationship between visual acuity and retinal thickness during anti-vascular endothelial growth factor therapy for retinal diseases. Am J Ophthalmol.

[64] Gerendas BS, Prager S, Deak G, Simader C, Lammer J, Waldstein SM (2018). Predictive imaging biomarkers relevant for functional and anatomical outcomes during ranibizumab therapy of diabetic macular oedema. Br J Ophthalmol.

[65] Alasil T, Keane PA, Updike JF, Dustin L, Ouyang Y, Walsh AC (2010). Relationship between optical coherence tomography retinal parameters and visual acuity in diabetic macular edema. Ophthalmology..

[66] Endo H, Kase S, Tanaka H, Takahashi M, Katsuta S, Suzuki Y (2021). Factors based on optical coherence tomography correlated with vision impairment in diabetic patients. Sci Rep.

[67] Kim BY, Smith SD, Kaiser PK (2006). Optical coherence tomographic patterns of diabetic macular edema. Am J Ophthalmol.

[68] Lai TT, Yang CM, Yang CH, Ho TC, Hsieh YT (2019). Treatment outcomes and predicting factors for diabetic macular edema treated with ranibizumab - One-year real-life results in Taiwan. J Formos Med Assoc.

[69] Li B, Zhang B, Chen Y, Li D (2020). Optical coherence tomography parameters related to vision impairment in patients with diabetic macular edema: A quantitative correlation analysis. J Ophthalmol.

[70] Santos AR, Gomes SC, Figueira J, Nunes S, Lobo CL, Cunha-Vaz JG (2014). Degree of decrease in central retinal thickness predicts visual acuity response to intravitreal ranibizumab in diabetic macular edema. Ophthalmologica..

[71] Zur D, Iglicki M, Busch C, Invernizzi A, Mariussi M, Loewenstein A (2018). OCT Biomarkers as functional outcome predictors in diabetic macular edema treated with dexamethasone implant. Ophthalmology..

[72] Starr MR, Salabati M, Mahmoudzadeh R, Patel LG, Ammar MJ, Hsu J (2021). Fluctuations in central subfield thickness associated with worse visual outcomes in patients with diabetic macular edema in clinical trial setting. Am J Ophthalmol.

[73] Wang VY, Kuo BL, Chen AX, Wang K, Greenlee TE, Conti TF (2022). Fluctuations in macular thickness in patients with diabetic macular oedema treated with anti-vascular endothelial growth factor agents. Eye.

[74] Kessler LJ, Auffarth GU, Bagautdinov D, Khoramnia R (2021). Ellipsoid zone integrity and visual acuity changes during diabetic macular edema therapy: a longitudinal study. J Diabetes Res.

[75] Kessler LJ, Bagautdinov D, Labuz G, Auffarth GU, Khoramnia R (2022). Semi-automated quantification of retinal and choroidal biomarkers in retinal vascular diseases: Agreement of spectral-domain optical coherence tomography with and without enhanced depth imaging mode. Diagnostics.

[76] Nagai N, Suzuki M, Uchida A, Kurihara T, Ban N, Minami S (2020). The area and number of intraretinal cystoid spaces predict the visual outcome after ranibizumab monotherapy in diabetic macular edema. J Clin Med.

